# Population genomics of *Digitaria insularis* from soybean areas in Brazil

**DOI:** 10.1002/ps.6577

**Published:** 2021-08-17

**Authors:** Acácio Gonçalves Netto, Erick MG Cordeiro, Marcelo Nicolai, Saul JP de Carvalho, Ramiro Fernando Lopez Ovejero, Caio ACG Brunharo, Maria I Zucchi, Pedro J Christoffoleti

**Affiliations:** ^1^ Crop Science Department Luiz de Queiroz College of Agriculture, University of São Paulo Piracicaba Brazil; ^2^ Agro do Mato Consulting, Santa Barbara D'Oeste São Paulo Brazil; ^3^ Federal Institute of Education, Science and Technology of the South of Minas Gerais Machado Minas Gerais Brazil; ^4^ Bayer Crop Science Brazil São Paulo Brazil; ^5^ Department of Crop and Soil Science Oregon State University Corvallis OR USA; ^6^ Secretariat of Agriculture and Food Supply of Sao Paulo State Piracicaba Brazil

**Keywords:** adaptation, admixture, glyphosate resistance, herbicide resistance, sourgrass, weed genomics

## Abstract

**BACKGROUND:**

*Digitaria insularis* is a weed species that has gained considerable importance in Brazil's soybean production areas that rely on glyphosate‐resistant cultivars. Herbicide‐resistant weed populations of this species have been reported in many regions in Brazil, first in the south, followed by later reports in the north. We hypothesized that the spread of herbicide‐resistant *D. insularis* is facilitated by movement of agricultural machinery from the southern regions of Brazil.

**RESULTS:**

Population genomics revealed a weak or no genetic structure (*F*_ST_ = [0; 0.16]), moderate expected heterozygosity (*H*_E_ = 0.15; 0.44) and low inbreeding (*F*_IS_ = [−0.1; 0.1]) in *D. insularis* populations. Our data supported the hypothesis that herbicide resistance gene flow predominantly occurred in a south‐to‐north direction based on a migration analysis. We also found evidence of local adaptation of resistant populations in the northern soybean‐growing regions of Brazil.

**CONCLUSION:**

Evidence in our work suggests that gene flow of glyphosate‐resistant *D. insularis* is associated with movement of agricultural machinery, although local selection pressure seems to play an important role in the evolution of herbicide resistance throughout the country. Our results suggest preventive practices such as equipment sanitation should be implemented to limit the spread of herbicide resistant *D. insularis*. © 2021 The Authors. *Pest Management Science* published by John Wiley & Sons Ltd on behalf of Society of Chemical Industry.

## Graphical abstract

An agricultural machine transporting *Digitaria insularis*across the country. This represents our results that glyphosate‐resistant *D. insularis* was likely dispersed *via* movement of agricultural machinery from the south to the north.

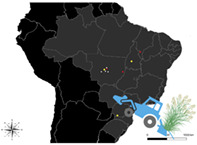

## 1 INTRODUCTION

Widespread adoption of herbicide‐resistant crops in agriculture has provided several benefits for society. For instance, more environmentally friendly compounds^1^ could be adopted that also provide adequate weed control and crop selectivity. Boosting agriculture production systems is one of the pillars of sustainable intensification of agriculture, and herbicides are essential to increase crop yields.^2^ However, the overreliance on herbicides as the primary weed management tool has selected for many herbicide‐resistant weed populations worldwide.^3^ To date, there are 514 unique cases of herbicide‐resistant weeds globally that have evolved resistance to 23 of the 26 known herbicide mechanisms of action.^3^

*Digitaria insularis* is a diploid (2*n* = 36), outcrossing,^4^ C_4_ perennial weed species native to South America that propagates by seed and rhizomes and is commonly found throughout the tropical regions of South, Central, and North America. The seeds have silky hairs that aid in long‐distance wind dispersal and attach to heavy machinery, whereas the rhizomes may aid dispersal following their fragmentation during agricultural practices (for example, tillage). Once plants become established, control is challenging due to the dual reproduction system this species exhibits. *D. insularis* is predominant in South America's crop‐growing regions, where the primary cropping system is a double‐crop year of soybean–corn followed by soybean–corn. Typically, corn and soybean varieties with glyphosate resistance traits are widely adopted during both crop seasons. Overreliance on glyphosate as the primary weed management tool selected for glyphosate‐resistant populations of *D. insularis* in Paraguay in 2005,^3^ and these are believed to have dispersed to Brazil soon after their first detection given their geographical proximity.^3^

In 2012, most of the glyphosate‐resistant populations were present in the southern states of Brazil (for example, São Paulo and Paraná). However, years later, many other glyphosate‐resistant populations were identified in the central and northern regions of Brazil.^5^ Recent studies have indicated that the dispersal of *D. insularis* throughout Brazil follows routes used in the movement of machinery (mainly combines and sprayers) from southern to northern regions of the country.^6^

This research aimed to investigate population genetic structure and gene flow among glyphosate‐resistant populations of *D. insularis* sampled in the most relevant soybean‐growing regions in Brazil. We hypothesize that alleles associated with glyphosate resistance have a single origin in southern Brazil and have dispersed northward aided by agricultural machinery and seasonal migration to the north. Understanding the sources of initial dispersal of herbicide‐resistant weed populations may help develop management practices and policies to contain herbicide resistance gene flow.

## 2 MATERIALS AND METHODS

### 2.1 Source of *D. insularis* populations

**Figure 1:**
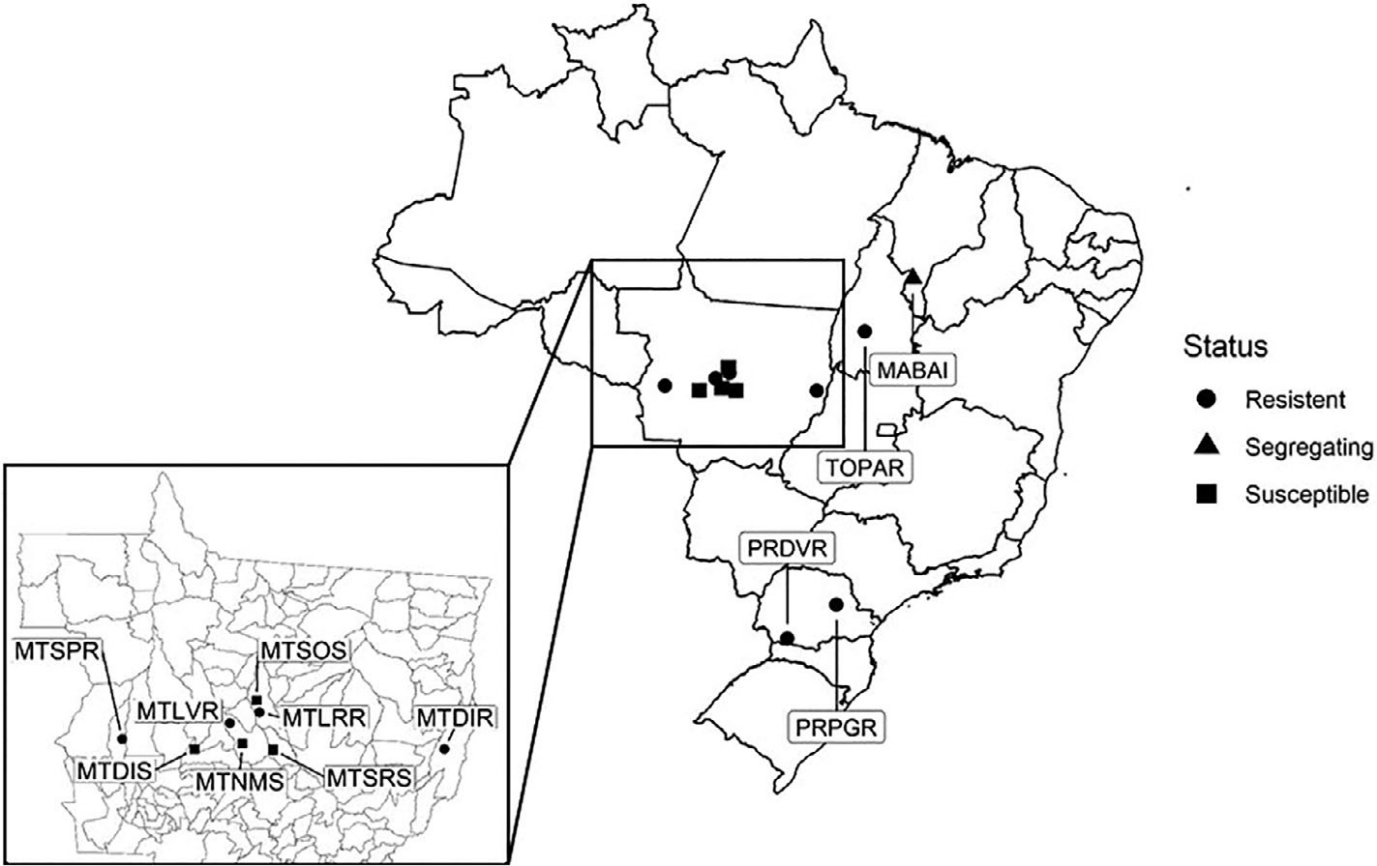
Origin of *Digitaria insularis* populations used in this study. Circles, diamonds and triangle represent glyphosate‐resistant, glyphosate‐susceptible and segregating, respectively.

*Digitaria insularis* specimens were sampled from 12 soybean farms in four different states in Brazil (Fig. 1 and Table S1). For each field, mature seed heads were randomly collected from 50 plants throughout the field, sealed in a paper bag, and stored in a dry environment until further analysis. Sampling took place late in the season, near completion of the soybean growing cycle. Therefore, sampled plants are likely escapees from the weed management practices of the current growing season. Seeds were germinated in commercial potting media, transplanted to 5 × 5 cm pots, and grown in a greenhouse for initial glyphosate resistance screenings. Three plants from each population were sprayed with 960 g acid equivalent (a.e.) ha^−1^ of glyphosate (Roundup Transorb R®) using Teejet XR11002 nozzles calibrated to deliver 200 L ha^−1^ of herbicide mixture. The response to glyphosate was assessed visually using a 0–100% scale, where 0% represents the absence of any visual symptoms and 100% represents complete plant death. Each population was classified as susceptible (if all plants exhibited more than 80% visual injury), segregating (if at least one plant exhibited more than 80% visual injury), and resistant (if all plants exhibited less than 80% visual injury). Only three individuals per population were screened because this step was part of a much larger project to map glyphosate resistance in *D. insularis*, and space constraints prevented a greater number of replications.

### 2.2 DNA extraction, quality control, sample preparation and sequencing

After the conclusion of the initial glyphosate resistance screening, seeds from the original field populations were germinated (eight plants per population). Leaf tissue was collected from the youngest fully expanded leaves on 10‐day‐old plants and immediately frozen in liquid nitrogen and stored in a −80°C freezer until further manipulation. After tissue collection, samples were treated with 960 g a.e. ha^−1^ of glyphosate to confirm the response to glyphosate for each individual in the populations. Glyphosate resistance status in this expanded screening agreed with the preliminary evaluation in Section 2.1.

Genomic DNA extraction was performed using the CTAB method,^7^ followed by a spectrophotometric quantification step using a NanoDrop (Thermo Scientific). A sample of DNA was digested with HindIII to assess DNA integrity by gel electrophoresis. The remainder of the sample was normalized to 30 ng μl^−1^ and digested with the restriction enzymes PstI and MseI (New England Biolabs).

The method proposed by Elshire *et al*.^8^ was adopted to prepare the libraries for genotype by sequencing (GBS). After DNA digestion, Illumina adapters with unique barcodes were ligated to the digested DNA, followed by pooling of 96 samples and amplification by polymerase chain reaction (PCR). The library was quantified with quantitative PCR (qPCR) using the KAPA Library Quantification kit (KAPA Biosystems) following the manufacturer's recommendations. Sequencing was performed in an Illumina NextSeq 500 in a single read mode (150 bp).

### 2.3 Genotypes and SNP filtering

Samples were demultiplexed and processed with the process_radtags module of Stacks v.2.437,^9^ considering the recognition sites of PstI and MseI, and single‐ended reads were truncated to 90 bp. The *de novo* pipeline in Stacks was adopted to first build loci de novo in *ustacks* using the parameters *M* = 2, *m* = 3 and *n* = 2, following the parameter optimization guidelines.^10^, ^11^ A catalog of loci was built with the *cstacks* module, followed by alignment of the reads to the catalog built using *sstacks*, and finally *gstacks* for genotype calling. The *populations* module filtered the dataset to keep loci that are common in at least 40% of the individuals in each population (*r* = 0.4), minor allele frequency of 5% (*–min‐maf *= 0.05), and maximum observed heterozygosity of 75% (*–max‐obs‐het *= 0.75). Outputs were saved in genepop, vcf, and structure (.str) formats for downstream analyses.

### 2.4 Determining loci under selection

We used BayeScan^12^ with default values to identify loci under neutral and positive selection. BayeScan uses a Markov Chain Monte Carlo method to estimate fixation indices for each population in the plant genome. A total of 20 pilot runs (*‐nbp* 20) with a length of 5000 (*‐pilot* 5000) were performed. We then performed a burn‐in of 50 000 (*‐burn* 50 000) interactions with 10 thinning intervals (*‐thin* 10). Prior odds for the neutral model was the default value (*‐pr_odds* 10) and posterior distribution of 0.95 as candidates for positive selection.

### 2.5 Genetic diversity, inbreeding coefficient and linkage disequilibrium

We used the R package adegenet^13^ to calculate the expected heterozygosity (*H*_E_) and inbreeding coefficient (*F*_IS_) based on the single nucleotide polymorphism (SNP) data set obtained. *F*_IS_ was calculated as 1 − (*H*_O_/*H*_E_), where *H*_O_ was the observed heterozygosity. Values near zero indicate random mating, whereas positive and negative values indicate inbreeding and outbreeding, respectively. We determined the indices of associations, ${\bar{r}}_{d}$,^14^ between any pair. The lack of association between pairs of loci indicates that markers are independent, which means that there has been recombination between the markers, whereas deviation from the expected genotypic frequencies can be interpreted as linkage. We used the R package poppr^15^ to analyze each population independently with 1000 permutations.

### 2.6 Fixation index, direction and magnitude of migration, and population structure

Relative migration between pairs of populations was calculated based on the allele frequency using the R package diveRsity,^16^ based on the *G*_ST_ statistics using 10 000 permutations to infer significance.^17^ This method builds relative migration levels between populations, and we included two separate analyses: (a) considering all potential migration networks, and (b) considering only those that are statistically significant based on non‐overlapping relative migration at the 95% confidence interval. The genetic structure was evaluated using the software ADMIXTURE v.1.22, where values of *K* were obtained from 1 to 15. The optimum *K* values were obtained using cross‐validation to infer the most probable number of ancestral populations.^18^ Principal component analysis (PCA) was performed with the R package adegenet^13^ and ade4.^19^ We performed independent PCA analyses with SNP exhibiting positive and neutral selection.

## 3 RESULTS

**Figure 2:**
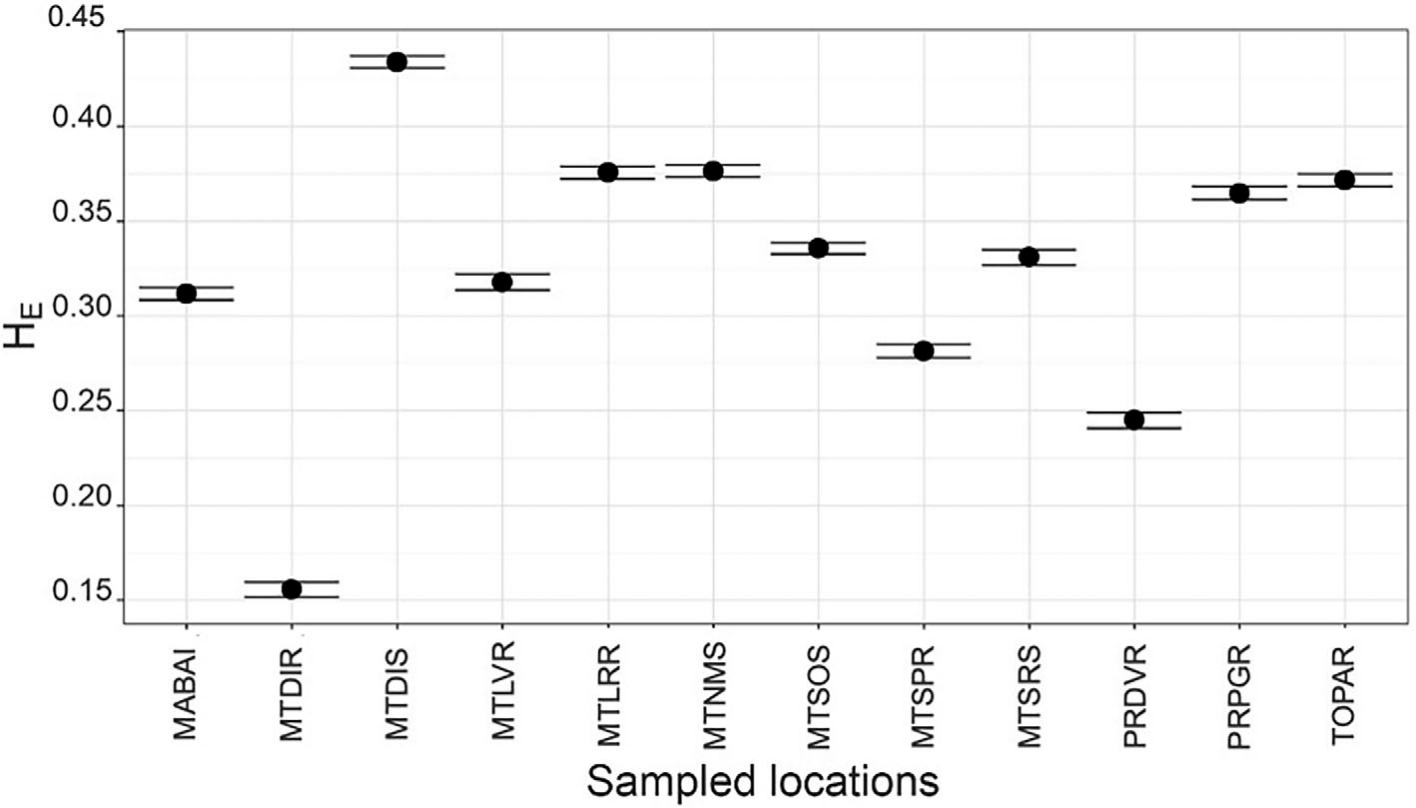
Observed heterozygosity (*H*_E_) for 12 *Digitaria insularis* populations from Brazil. Solid black circles represent mean values, whereas bars represent 95% confidence intervals. For population origins and labels, see Figs 1 and S1.

**Figure 3:**
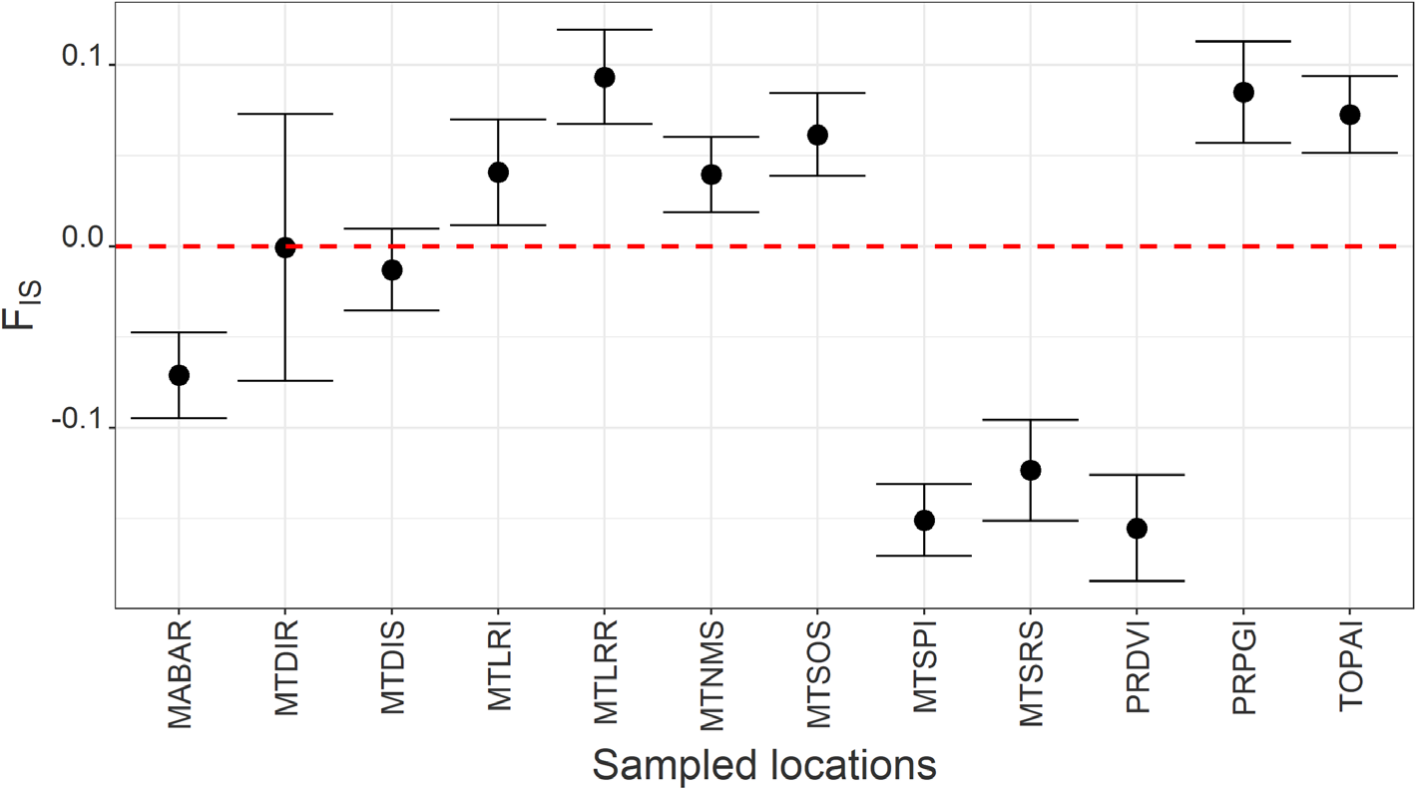
Inbreeding coefficient (*F*_IS_) calculated for 12 *Digitaria insularis* populations from Brazil. Solid black circles represent mean values, whereas bars represent 95% confidence intervals. The dashed red line represents the zero *F*_IS_ value. For population origins and labels, see Figs 1 and S1.

In total, 4245 SNPs were generated using Stacks and were subsequently used for the population genomics analysis in this study. Considerable variation in *H*_E_ was observed among the sampled populations (Fig. 2). The populations with the highest and lowest *H*_E_ values were MTDIS and MTDIR, respectively. Originating from Paraná state, PRDVR exhibited the lowest *H*_E_ value. *F*_IS_ values also ranged widely (Fig. 3). Four populations from distinct locations had negative *F*_IS_ values (populations below red, dashed line), whereas six locations had positive *F*_IS_ values. Two populations had *F*_IS_ values equal to zero.

Average *F*_ST_ values varied from 0 to 0.16, indicating low to moderate differentiation (Table 1). Population MABAI, from northern Brazil (Maranhão state), had the highest *F*_ST_ value (0.16) compared with population MTSOS. Low genetic structure was observed for most pairwise comparisons indicating high gene flow. Unrooted phylogenetic analysis (Fig. S1) supports *F*_ST_ analysis because the radial layout of the tree indicates a close genetic relationship among the collected *D. insularis* locations.

**Table 1:** Pairwise fixation index (F_ST_) (upper) and *P*‐values (lower) for 12 *Digitaria insularis* populations from Brazil

	MABAI*	MTDIR	MTDIS	MTLVR	MTLRR	MTNMS	MTSOS	MTSPR	MTSRS	PRDVR	PRPGR	TOPAR
MABAI		−0.46	**0.05**	**0.08**	**0.07**	**0.07**	**0.16**	**0.05**	**0.11**	−0.06	**0.08**	−0.02
MTDIR	1.00		−0.99	−0.63	−0.46	−0.70	−0.45	−0.53	−0.67	−0.61	−0.69	−0.89
MTDIS	0.01	1.00		**0.02**	**0.05**	−0.03	−0.08	−0.10	−0.06	−0.15	−0.01	**0.04**
MTLVR	0.00	1.00	0.02		**0.10**	**−0.01**	**0.07**	**0.06**	−0.07	**0.01**	**0.11**	**0.06**
MTLRR	0.00	1.00	0.00	0.00		**0.07**	**0.10**	−0.03	**0.02**	−0.07	**0.05**	**0.07**
MTNMS	0.00	1.00	0.44	0.06	0.00		−0.02	−0.05	−0.06	−0.10	0.02	0.03
MTSOS	0.00	1.00	0.95	0.00	0.00	0.18		**0.06**	−0.06	**0.07**	**0.03**	**0.07**
MTSPR	0.00	1.00	0.95	0.00	0.43	0.51	0.01		0.01	−0.05	−0.03	−0.12
MTSRS	0.00	1.00	0.87	0.76	0.02	0.72	0.63	0.07		−0.06	−0.02	0.01
PRDVR	0.64	1.00	0.99	0.02	0.82	0.94	0.01	0.41	0.51		−0.04	−0.14
PRPGR	0.00	1.00	0.10	0.00	0.00	0.00	0.00	0.32	0.19	0.52		0.05
TOPAR	0.20	1.00	0.00	0.00	0.00	0.01	0.00	0.96	0.06	1.00	0.00	

^*^ Values in bold represent F_ST_ values different than zero (*P* < 0.05). For population origins, see Fig. S1.

The ${\bar{r}}_{d}$ coefficients were significant (*P* < 0.001) in 7 of the 12 sampled locations, indicating disequilibrium between markers in those locations (Table 2). In this test, the null hypothesis is that no linkage exists between markers, and consequently sexual reproduction is predominant. Samples from Paraná (PRDVR and PRPGR), Maranhão (MABAI) and Mato Grosso state (MTLVR and MTSPR) seem to be in equilibrium, whereas MTDIR, MTDIS, MTLRR, MTNMS, MTSOS and MTSRS (from Mato Grosso state), as well as TOPAR (from Tocantins state) are in disequilibrium (Table 2).

**Table 2:** Index of association in 12 *Digitaria insularis* populations in Brazil

Sample ID^*^	Indices of association (${\bar{r}}_{d})$	*P*‐value
MABAI	0.0775	1.000
MTDIR	0.0833	0.001
MTDIS	0.0289	0.001
MTLVR	0.0415	1.000
MTLRR	0.0602	0.001
MTNMS	0.0356	0.001
MTSOS	0.0755	0.001
MTSPR	0.0666	1.000
MTSRS	0.0871	0.001
PRDVR	0.0356	1.000
PRPGR	0.0575	0.496
TOPAR	0.0175	0.001

^*^ For population origins, see Fig. S1.

**Figure 4:**
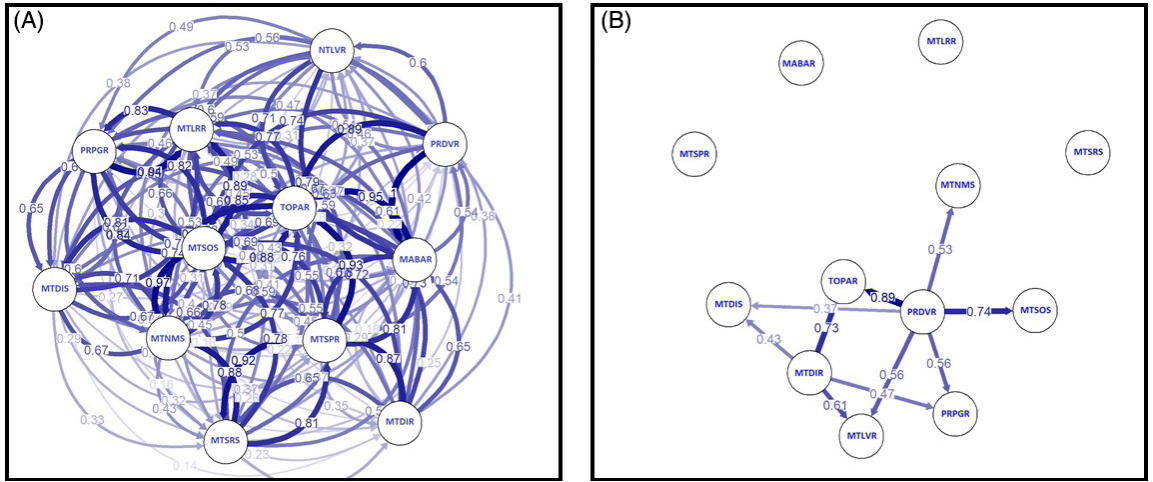
Direction of gene flow and magnitude of migration in *Digitaria insularis* populations from Brazil using *G*_ST_. Arrows indicate the direction of gene flow; numbers represent the relative coefficient of migration. (A) All potential migration routes. (B) Migration routes after a threshold filter is implemented of 0.05 probability.

An asymmetric direction of dispersal was observed in *D. insularis*, driven primarily by populations PRDVR and MTDIR (Fig. 4 ). Population PRDVR contributed most to the number of migrants and exhibited the largest impact on the population dynamics of this weed in the sampled populations from Brazil. Population MTDIR was the second most influential population and contributed to alleles in Mato Grosso, Paraná and Tocantins states. Other populations showed less involvement in the dispersal of alleles throughout the country. Interestingly, of the southern populations, only PRDVR showed patterns of migration to other *D. insularis* populations; by contrast PRPGR did not show significant migration networks (Fig. 4(B)). If our hypothesis is correct that gene flow in *D. insularis* is primarily mediated by the movement of agricultural machinery, then local agricultural practices (for example, tillage versus no‐tillage, cropping system, rotation sequence), as well as farm ownership will play an important role in the direction of gene flow.

**Figure 5:**
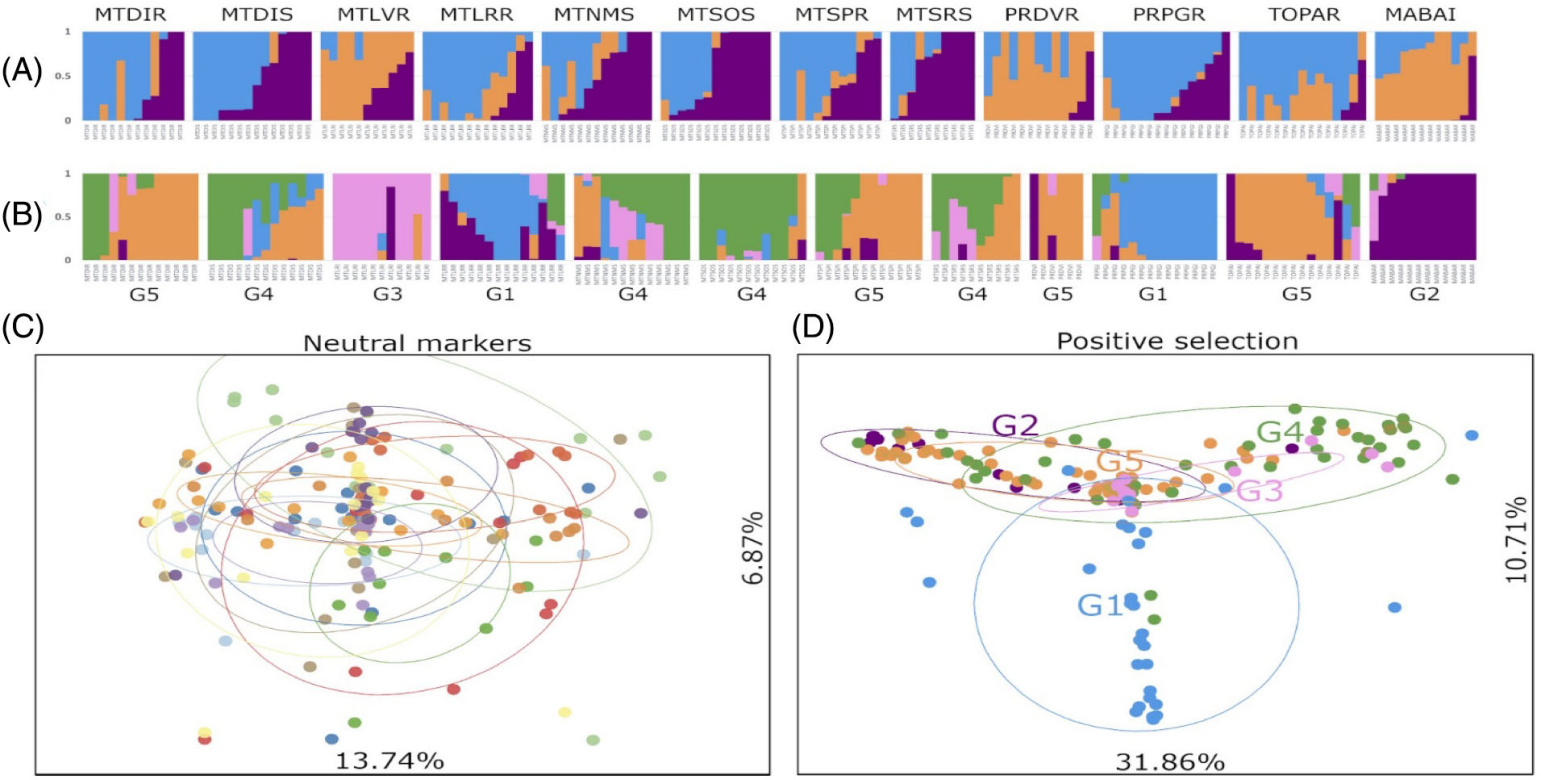
ADMIXTURE and principal component analysis (PCA) of 12 *Digitaria insularis* populations. ADMIXTURE analysis was performed using 1134 neutral single nucleotide polymorphisms (SNPs) with *K* = 3 (A), and 687 SNPs under positive selection and *K* = 5 (B). PCA with neutral SNPs (C) and SNPs under positive selection (D) were also performed. Populations were divided into five groups (G1–G5) for the analyses with SNPs under positive selection.

In total, 1134 SNPs were putatively neutral, and 687 had evidence of positive selection, according to BayeScan analysis. ADMIXTURE analysis using the 1134 neutral SNPs indicated that *K* = 3 was the most appropriate number of clusters given the populations under study (Fig. 5(A)) based on the cross‐validation test. Limited population structure was observed in most collected regions, except MABAI, PRDVR and MTLVR.

When we used only SNPs under positive selection, a clearer clustering pattern could be observed. Structure analyses using markers putatively under selection revealed clustering patterns compatible with geographical location and resistance status (*K* = 5). Using ADMIXTURE results on markers putatively under positive selection, we proposed grouping labels to aid visualization: G1, MTLRR and PRPGR (blue); G2, MABAI (purple); G3, MTLVR (pink); G4, MTNMS, MTSOS and MTSRS (green); and G5, MTDIR, MTSPR, PRDVR and TOPAR (orange). Locations sharing the same resistance status within the same broad geographical location tended to share more ancestry and be more closely grouped. A similar pattern was observed in the susceptible samples.

PCA broadly supports the conclusions of the ADMIXTURE analysis showing no structure when the neutral markers were used but more evident clustering when positive markers were used (Fig. 5(C),(D)). The three groups containing resistant individuals (G1, G3 and G5) were separated from one another. PC1 explained a significant variation (13.74 to 31.86%) as did PC2 (6.87 to 10.71%) for both SNP data sets. This pattern is further corroborated by phylogenetic and *F*_ST_ analyses.

## 4 DISCUSSION

*Digitaria insularis* is considered one of the most troublesome weed species in Brazil's soybean cropping systems, particularly aggravated by the widespread evolution of glyphosate‐resistant populations. Currently, two hypotheses can explain the rapid pace with which resistance has evolved over an extensive geographic range. The first hypothesis suggests that resistance evolved once in the south, where it was first reported in Paraguay, and then spread northwards. The second hypothesis suggests that resistance to glyphosate evolved multiple times. Our data indicate that one of the main features of *D. insularis* population dynamics is the high degree of gene flow between areas, which makes the first hypothesis more likely. However, using only putative markers under positive selection, three different clusters containing resistant samples could be identified, suggesting a different pattern of evolution in the various regions.

ADMIXTURE analysis reveals a moderate genetic structure between the two southern locations (PRDVR and PRPGR), but both locations in the south seem to be well connected to Mato Grosso's central locations. These results may support the hypothesis that movement of agricultural machinery could have assisted the spread of *D. insularis* to the north. In general, soybean sowing occurs earlier in southern regions than in the north due to precipitation patterns. Therefore, machinery becomes available for use in Brazil's northern regions later in the season after operations in the south are finished. This sharing of agricultural equipment is only possible because many farmers own land in both locations and because third‐party companies in different regions may perform custom farming operations. Preventing weed propagule movement is one of the pillars of integrated weed management, and sanitizing machinery is crucial.^20^, ^21^ Our results support the notion that long‐distance spread of *D. insularis* followed the direction of the agricultural machinery movement and could be used to better inform farmers and agronomists of the importance of sanitizing equipment.

Considering the timeline over which glyphosate resistance has developed in South America, we can relate our results to historical facts. Population PRDVR was collected from south Paraná state, near the border with Paraguay, where the first glyphosate‐resistant *D. insularis* was reported. Unfortunately, we did not include the Paraguayan population in our analysis, but the high gene flow observed in this study might suggest that resistance genes have spread across the borders. Movement of *D. insularis* propagules between countries is not unexpected because many farmers own land on both sides of the border.

Overall, no clear relation between the observed *H*_E_ (genetic diversity) and glyphosate resistance status or geographic region can be drawn. For instance, MTDIR exhibited the lowest *H*_E_, whereas MTLRR exhibited one of the largest values. These results indicated that local *D. insularis* populations might be involved in the evolutionary rescue of glyphosate‐resistant populations, increasing *H*_E_ after the initial bottleneck caused by the herbicide.^22^ This is because once a glyphosate‐resistant population is introduced to a new location, genetic diversity is limited because of the few individuals that founded the population. However, because of the outcrossing mating system of *D. insularis*, genetic diversity may be restored by gene flow from other localities. This may also enable populations to adapt more rapidly to the management practices other crop production systems.^23^ Our *H*_E_ results indicate that the magnitude of *D. insularis* dispersion is greater than observed in the outcrossing weed species *Alopecurus myosuroides*, where it was found that *H*_E_ varied from 0.09 to 0.14, approximately, in populations exhibiting different patterns of herbicide resistance.^24^ Similarly, the *H*_E_ value of *D. insularis* is larger than that of the predominantly self‐pollinated weed species *Bromus tectorum* which exhibited a mean *H*_E_ of 0.2.^25^ The higher *H*_E_ levels in *D. insularis* further support the idea that admixture plays an important role in maintaining genetic diversity in populations.

Estimates of the inbreeding coefficient (*F*_IS_) indicated that populations exhibited limited inbreeding because these values ranged between −0.1 and 0.1, suggesting the *D. insularis* populations studied maintained their outcrossing behavior. The observed low inbreeding coefficients could indicate the limited ability of *D. insularis* to self‐pollinate under local environmental conditions. For example, in *Lolium multiflorum*, an outcrossing weed species, *F*_IS_ ranged from 0.374 to 0.475 for 14 glyphosate‐resistant populations from California.^26^ Asexual reproduction in populations of *D. insularis* was particularly important for 7 of the 12 populations studied, according to the index of association analysis (Table 2), corroborating the *F*_IS_ results.

BayeScan analysis identified more than1000 loci under selection. It is difficult to infer whether the SNPs are physically close because of the absence of a reference genome for this species, although there are ongoing efforts to make this resource available.^27^ Therefore, it is possible that a few regions of the genome are under selection. Furthermore, BayeScan analysis may also identify selection pressure from other agents, such as use of other herbicides, management practices and climate conditions.

Populations were collected in glyphosate‐resistant soybean areas, where multiple herbicide applications were likely made before sampling. To further eliminate glyphosate‐susceptible genotypes, we applied a lethal glyphosate dose to individuals to ensure the genotype‐by‐sequencing study was conducted with known resistant and susceptible individuals. Interestingly, at least two different genetic clusters of resistance are apparent when the resistant populations are considered. For instance, resistant population PRPGR exhibits, at *K* = 3, individuals that are entirely from the blue and purple backgrounds. Because they are all glyphosate resistant, the resistance alleles are both found in the blue and purple genetic backgrounds. This suggests that glyphosate resistance might interplay with different regional dynamics, including multiple mutations in different genetic backgrounds that likely evolved multiple times independently. More research needs to be conducted to identify whether these mutations are similar among populations. The mechanisms of glyphosate resistance in *D. insularis* have not been completely elucidated. Glyphosate‐resistant *D. insularis* populations are characterized by not exhibiting mutations in the gene that encoded glyphosate's target enzyme; however, they may prevent the herbicide from systemically moving in the plant.^28^, ^29^ Conversely, other resistant populations do not exhibit target site mutations or reduced herbicide translocation,^29^ indicating non‐target‐site resistance mechanisms are predominant in *D. insularis* and will require more integrated ‘‐omics’ approaches to improve our understanding.^30^

## 5 CONCLUSION

Here, we uncover important aspects of *D. insularis* population dynamics in Brazilian soybean fields. Outcrossing populations spread their genes across a large range likely aided by heavy machinery. *D. insularis* populations are under strong positive selection associated with herbicide usage; however, clustering patterns suggest subtle differences in the process of resistance evolution in different areas. Future research should address two main follow‐up questions. What are the mechanisms of resistance in these populations? Moreover, did glyphosate resistance in the *D. insularis* populations studied here evolve the same resistance mechanisms (but in different genetic backgrounds)? Answering these questions will help weed scientists develop better predictive models and understand how selection pressure by herbicides shapes weed populations and the best management practices to slow the evolution of herbicide resistance.^31^

## Supporting information


**FIGURE S1** Unrooted phylogenetic tree. Circles represent individual populations, colored by population. Red: MABAI. Gray: MTLVR, MTLRR, MTSPR, MTSRS, MTDIS, MTSOS, MTNMS, MTDIR. Blue: PRDVR. Purple: PRPGR. Green: TOPAR.

**TABLE S1** *Digitaria insularis* population origins and glyphosate resistance status.

**TABLE S2** Genetic differentiation (*G*_ST_) values representing amount of gene flow from populations in the first column to populations in the first row. For instance, population PRDVR to TOPAR exhibits the greatest gene flow that is statistically significant. Bold numbers indicate statistical significance based on 95% bootstrapping confidence intervals.
