# Supplementary figures and images for: Population genomics of Digitaria insularis from soybean areas in Brazil
Source: Pest Manag Sci. 2021 Aug 17;77(12):5375–81. doi: 10.1002/ps.6577 (PMC9291757; doi:10.1002/ps.6577)

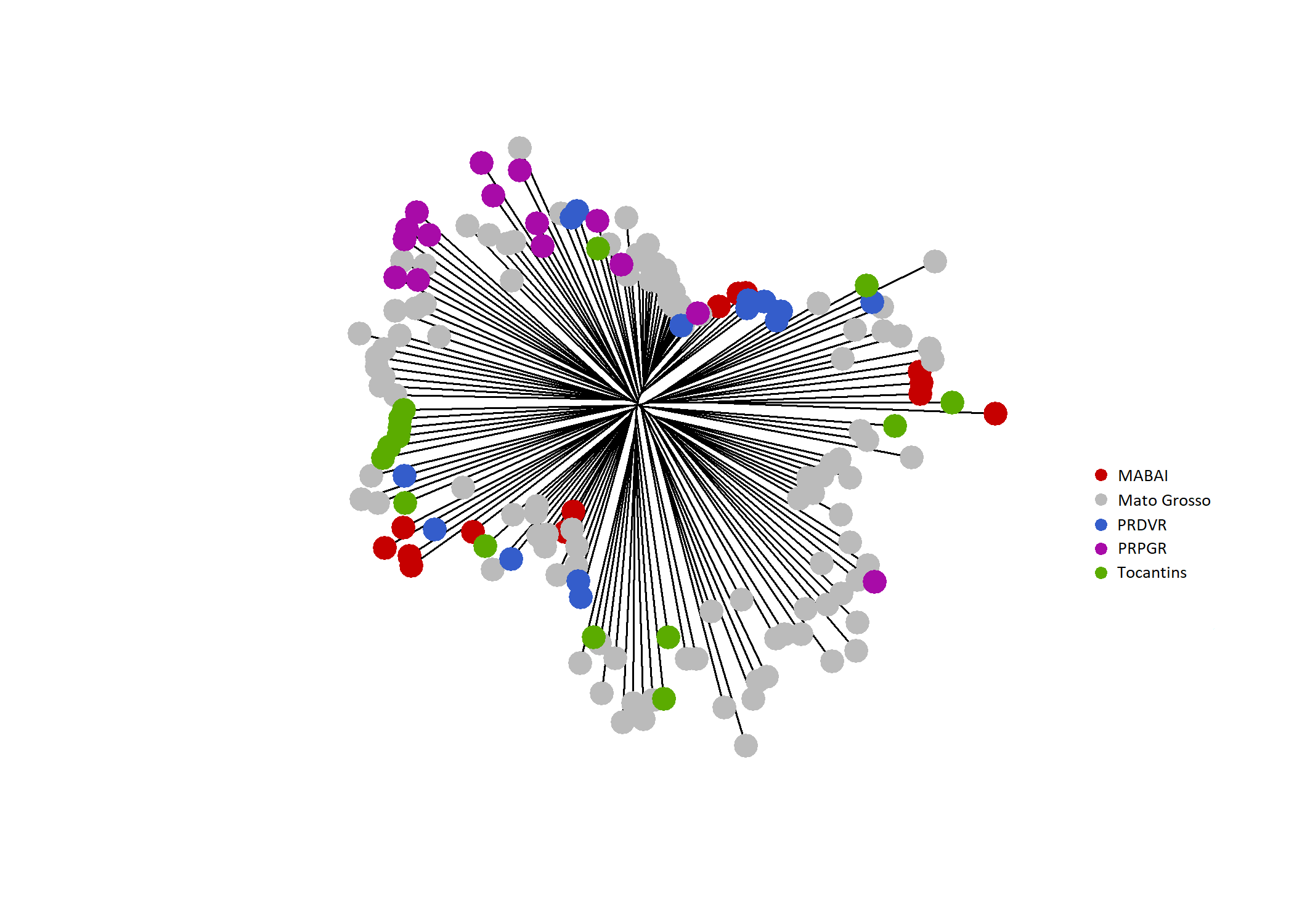

Supplement: Supplementary file 1 — FIGURE S1 Unrooted phylogenetic tree. Circles represent individual populations, colored by population. Red: MABAI. Gray: MTLVR, MTLRR, MTSPR, MTSRS, MTDIS, MTSOS, MTNMS, MTDIR. Blue: PRDVR. Purple: PRPGR. Green: TOPAR. [file PS-77-5375-s003.jpeg]
