# Supplementary material for: Population genomics of Digitaria insularis from soybean areas in Brazil
Source: Pest Manag Sci. 2021 Aug 17;77(12):5375–81. doi: 10.1002/ps.6577 (PMC9291757; doi:10.1002/ps.6577)
Supplement: Supplementary file 2 — TABLE S1 Digitaria insularis population origins and glyphosate resistance status. [file PS-77-5375-s002.docx]

Table S1. *Digitaria insularis* population origins and glyphosate resistance status.

| **Sample ID** | **State** | **Resistance Status** | **Latitude** | **Longitude** |
| --- | --- | --- | --- | --- |
| PRDVR | Paraná | Resistant | -26.100278 | -52.862778 |
| PRPGR | Paraná | Resistant | -24.433889 | -50.353889 |
| TOPAR | Tocantins | Resistant | -10.926667 | -48.900000 |
| MABAI | Maranhão | Segregating | -8.288889 | -46.468611 |
| MTLVR | Mato Grosso | Resistant | -13.233889 | -56.510000 |
| MTLRR | Mato Grosso | Resistant | -12.981389 | -55.803056 |
| MTSPR | Mato Grosso | Resistant | -13.606944 | -59.101667 |
| MTSRS | Mato Grosso | Susceptible | -13.853611 | -55.479444 |
| MTDIS | Mato Grosso | Susceptible | -13.843333 | -57.358056 |
| MTSOS | Mato Grosso | Susceptible | -12.705000 | -55.868056 |
| MTNMS | Mato Grosso | Susceptible | -13.714722 | -56.205000 |
| MTDIR | Mato Grosso | Resistant | -13.843333 | -51.358056 |
