# Supplementary material for: Population genomics of Digitaria insularis from soybean areas in Brazil
Source: Pest Manag Sci. 2021 Aug 17;77(12):5375–81. doi: 10.1002/ps.6577 (PMC9291757; doi:10.1002/ps.6577)
Supplement: Supplementary file 3 — TABLE S2 Genetic differentiation (G ST) values representing amount of gene flow from populations in the first column to populations in the first row. For instance, population PRDVR to TOPAR exhibits the greatest gene flow that is statistically significant. Bold numbers indicate statistical significance based on 95% bootstrapping confidence intervals. [file PS-77-5375-s001.docx]

Table S2. Genetic differentiation (G_ST_) values representing amount of gene flow from populations in the first column to populations in the first row. For instance, population PRDVR to TOPAR exhibits the greatest gene flow that is statistically significant. Bold numbers indicate statistical significance based on 95% bootstrapping confidence intervals.

| Populations | | 1 | 2 | 3 | 4 | 5 | 6 | 7 | 8 | 9 | 10 | 11 | 12 |
| --- | --- | --- | --- | --- | --- | --- | --- | --- | --- | --- | --- | --- | --- |
| 1 | MABAR |  | 0.287 | 0.493 | 0.415 | 0.538 | 0.689 | 0.668 | 0.803 | 0.585 | 0.542 | 0.468 | 0.955 |
| 2 | MTDIR | 0.652 |  | **0.434** | 0.381 | **0.608** | 0.550 | 0.718 | 0.872 | 0.647 | 0.406 | **0.469** | **0.726** |
| 3 | MTDIS | 0.244 | 0.142 |  | 0.216 | 0.745 | 0.669 | 0.673 | 0.315 | 0.329 | 0.121 | 0.574 | 0.455 |
| 4 | MTLRR | 0.792 | 0.186 | 0.381 |  | 0.526 | 0.464 | 0.605 | 0.449 | 0.302 | 0.457 | 0.487 | 0.713 |
| 5 | MTLVR | 0.314 | 0.155 | 0.670 | 0.306 |  | 0.621 | 0.662 | 0.311 | 0.268 | 0.161 | 0.831 | 0.686 |
| 6 | MTNMS | 0.368 | 0.232 | 0.712 | 0.322 | 0.690 |  | 0.781 | 0.365 | 0.433 | 0.181 | 0.533 | 0.765 |
| 7 | MTSOS | 0.448 | 0.261 | 0.806 | 0.368 | 0.887 | 0.970 |  | 0.500 | 0.416 | 0.194 | 0.815 | 0.877 |
| 8 | MTSPR | 0.812 | 0.354 | 0.532 | 0.420 | 0.629 | 0.682 | 0.765 |  | 0.919 | 0.541 | 0.500 | 0.934 |
| 9 | MTSRS | 0.508 | 0.321 | 0.664 | 0.355 | 0.553 | 0.883 | 0.782 | 0.810 |  | 0.251 | 0.456 | 0.574 |
| 10 | PRDVR | 1.000 | 0.242 | **0.373** | 0.600 | **0.564** | **0.532** | **0.738** | 0.592 | 0.335 |  | **0.557** | **0.894** |
| 11 | PRPGR | 0.291 | 0.160 | 0.648 | 0.283 | 0.941 | 0.604 | 0.842 | 0.336 | 0.290 | 0.142 |  | 0.748 |
| 12 | TOPAR | 0.521 | 0.222 | 0.654 | 0.368 | 0.775 | 0.897 | 0.849 | 0.413 | 0.403 | 0.225 | 0.591 |  |
